# Cordycepin Suppresses Endothelial Cell Proliferation, Migration, Angiogenesis, and Tumor Growth by Regulating Focal Adhesion Kinase and p53

**DOI:** 10.3390/cancers11020168

**Published:** 2019-02-01

**Authors:** Yi-Ting Lin, Shu-Man Liang, Ya-Ju Wu, Yi-Ju Wu, Yi-Jhu Lu, Yee-Jee Jan, Bor-Sheng Ko, Yung-Jen Chuang, Song-Kun Shyue, Cheng-Chin Kuo, Jun-Yang Liou

**Affiliations:** 1 Institute of Cellular and System Medicine, National Health Research Institutes, Zhunan 350, Taiwan; 014260@nhri.org.tw (Y.-T.L.); shu-man@nhri.org.tw (S.-M.L.); kathy614@livemail.tw (Y.-J.W.); yijhu@nhri.org.tw (Y.-J.L.); kuocc@nhri.org.tw (C.-C.K.); 2 Institute of Bioinformatics and Structure Biology, National Tsing Hua University, Hsinchu 300, Taiwan; yjchuang@life.nthu.edu.tw; 3 Department of Pathology, Taichung Veterans General Hospital, Chiayi Branch, Chiayi City 600, Taiwan; werepast@gmail.com; 4 Department of Pathology and Laboratory Medicine, Taichung Veterans General Hospital, Taichung 407, Taiwan; yejan@vghtc.gov.tw; 5 Department of Internal Medicine, National Taiwan University Hospital, Taipei 100, Taiwan; kevinkomd@gmail.com; 6 Department of Medical Science, National Tsing Hua University, Hsinchu 300, Taiwan; 7 Institute of Biomedical Sciences, Academia Sinica, Taipei 115, Taiwan. skshyue@ibms.sinica.edu.tw; 8 Graduate Institute of Biomedical Sciences, China Medical University, Taichung 404, Taiwan

**Keywords:** angiogenesis, cordycepin, endothelial cells, FAK, p53

## Abstract

Focal adhesion kinase (FAK) plays an important role in vascular development, including the regulation of endothelial cell (EC) adhesion, migration, proliferation, and survival. 3’-deoxyadenosine (cordycepin) is known to suppress FAK expression, cell migration, and the epithelial–mesenchymal transition in hepatocellular carcinoma (HCC). However, whether cordycepin affects FAK expression and cellular functions in ECs and the specific molecular mechanism remain unclear. In this study, we found that cordycepin suppressed FAK expression and the phosphorylation of FAK (p-FAK) at Tyr397 in ECs. Cordycepin inhibited the proliferation, wound healing, transwell migration, and tube formation of ECs. Confocal microscopy revealed that cordycepin significantly reduced FAK expression and decreased focal adhesion number of ECs. The suppressed expression of FAK was accompanied by induced p53 and p21 expression in ECs. Finally, we demonstrated that cordycepin suppressed angiogenesis in an in vivo angiogenesis assay and reduced HCC tumor growth in a xenograft nude mice model. Our study indicated that cordycepin could attenuate cell proliferation and migration and may result in the impairment of the angiogenesis process and tumor growth via downregulation of FAK and induction of p53 and p21 in ECs. Therefore, cordycepin may be used as a potential adjuvant for cancer therapy.

## 1. Introduction

Angiogenesis is a crucial process in embryonic development, organogenesis, and tumor progression. Vascular endothelial cells (ECs) play a vital role in angiogenesis, enhanced EC migration and proliferation [1]. Focal adhesion kinase (FAK) is a cytoplasmic tyrosine kinase that contributes to the regulation of cell adhesion, spreading, migration, and survival [2,3,4]. An earlier study reported that a deficiency in FAK could reduce cell motility and enhance focal adhesion contact formation in mesodermal cells [5]. The conditional knockout of FAK in ECs could lead to aberrant lamellipodial extensions, resulting in cytoskeleton changes and reducing EC tubulogenesis, survival, proliferation, and migration [6,7]. Vascular ECs derived from FAK transgenic mice have been reported to promote angiogenesis compared with those derived from wild-type mice [8]. Moreover, the results of a study using endothelium-specific inducible FAK knockout mice revealed that tumor growth and tumor-induced angiogenesis were attenuated in mice with FAK deletion [9]. These results suggest that the expression of FAK plays a crucial role in the regulation of vascular development and tumor angiogenesis [6,7,8,9]. 

The promoter region of FAK harbors NFκB- and p53-binding sites [10]. Activation of NFκB and p53 can induce and suppress, respectively, FAK promoter activity [10]. Silencing of p53 or introduction of p53 functional mutants can result in increased FAK expression [11,12]. An earlier study reported that FAK plays an important role in the negative regulation of p53. FAK-transduced extracellular matrix survival signals have been found to impair p53-dependent apoptosis [13]. Interestingly, the N-terminal domain of FAK directly interacts with the N-terminal transactivation domain of p53, and FAK and p53 are co-localized in both the cytoplasm and nucleus [14]. In addition, accumulation of nuclear FAK promotes cell proliferation and survival by enhancing Mdm2-dependent p53 ubiquitination and degradation [15]. These findings suggest that the reciprocal interactions between FAK and p53 are regulated in a negative feedback manner.

Cordycepin (3-deoxyadenosine, Figure S1), a major ingredient found in the related fungus-infected caterpillars of *Cordyceps militaris*, has been suggested to possess anti-tumor properties in various types of malignancies [16,17,18,19,20,21,22,23,24,25,26,27,28,29,30]. A previous study reported that cordycepin could suppress integrin/FAK expression, the epithelial–mesenchymal transition (EMT), and cell migration in HCC [17]. In ECs, the expression of FAK is crucial for regulating various cellular functions. However, whether cordycepin affects FAK and related signaling factors remains unclear. In this study, we aimed to investigate the effect of cordycepin on tumor angiogenesis and elucidate the specific molecular mechanism. We showed for the first time that cordycepin could attenuate cell proliferation, migration, angiogenesis, and tumor growth via downregulation of FAK and induction of p53 and p21 in ECs. Therefore, cordycepin may be used as a potential supplement for anti-angiogenic therapy when combined with anti-tumor drugs.

## 2. Results

### 2.1. Suppression of FAK Expression in ECs by Cordycepin

**Figure 1:**
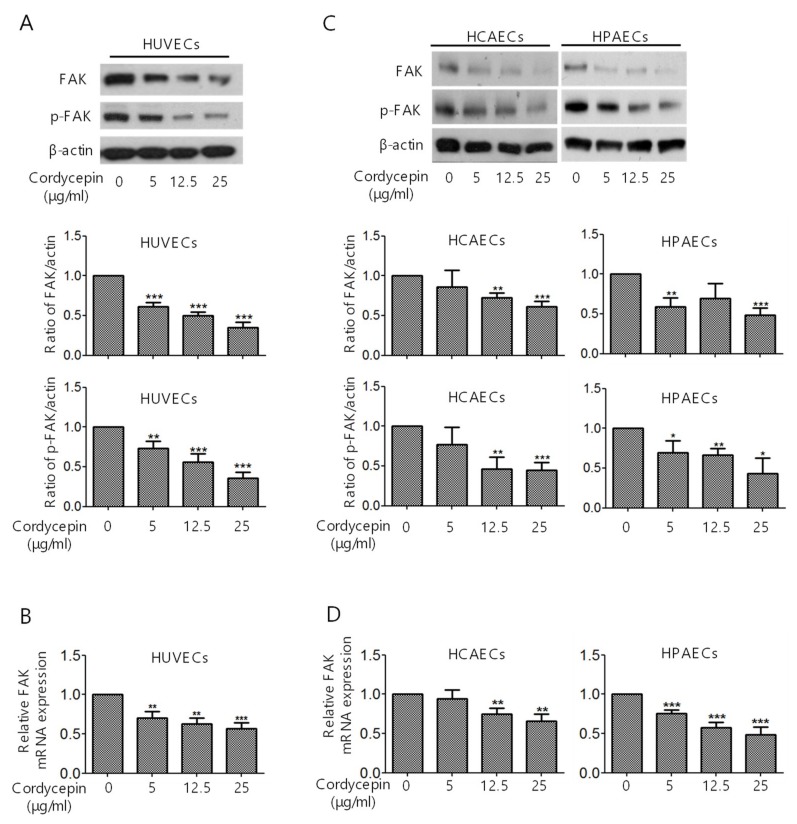
Suppression of focal adhesion kinase (FAK) expression and FAK phosphorylation in endothelial cells (ECs) by cordycepin. (**A,C**) HUVECs, HCAECs, and HPAECs were treated with 0–25 μg/mL cordycepin for 24 h, and FAK and p-FAK expression levels were determined by western blotting analysis. (**B,D**) HUVECs, HCAECs, and HPAECs were treated with 0–25 μg/mL cordycepin for 4 h, and the expression level of FAK was determined by qPCR. β-actin was used as the loading control. Scale bars: mean ± SD. * *p* < 0.05, **, *p* < 0.01; ***, *p* < 0.001.

To investigate whether cordycepin regulates FAK expression in ECs, human umbilical vein endothelial cells (HUVECs) were treated with cordycepin (0–25 μg/mL) for 24 h. The protein expression levels of FAK and p-FAK were evaluated by western blotting analysis. We found that cordycepin significantly suppressed FAK expression in a dose-dependent manner, which was accompanied by a reduction in p-FAK (Figure 1A). The suppression of FAK mRNA expression in HUVECs was also demonstrated by qPCR analysis (Figure 1B). To further confirm the suppression of FAK/p-FAK by cordycepin in ECs, we examined the expression levels of FAK and p-FAK in human coronary artery endothelial cells (HCAECs) and human pulmonary artery endothelial cells (HPAECs). HCAECs and HPAECs were treated with cordycepin. Similar to its effect on HUVECs, cordycepin reduced FAK and p-FAK levels, as demonstrated by western blotting (Figure 1C: left panels, HCAECs; right panels, HPAECs) and qPCR (Figure 1D: left panel, HCAECs; right panel, HPAECs) analysis.

### 2.2. Inhibition of EC Migration, Proliferation, Tube Formation, and In Vivo Angiogenesis by Cordycepin

**Figure 2:**
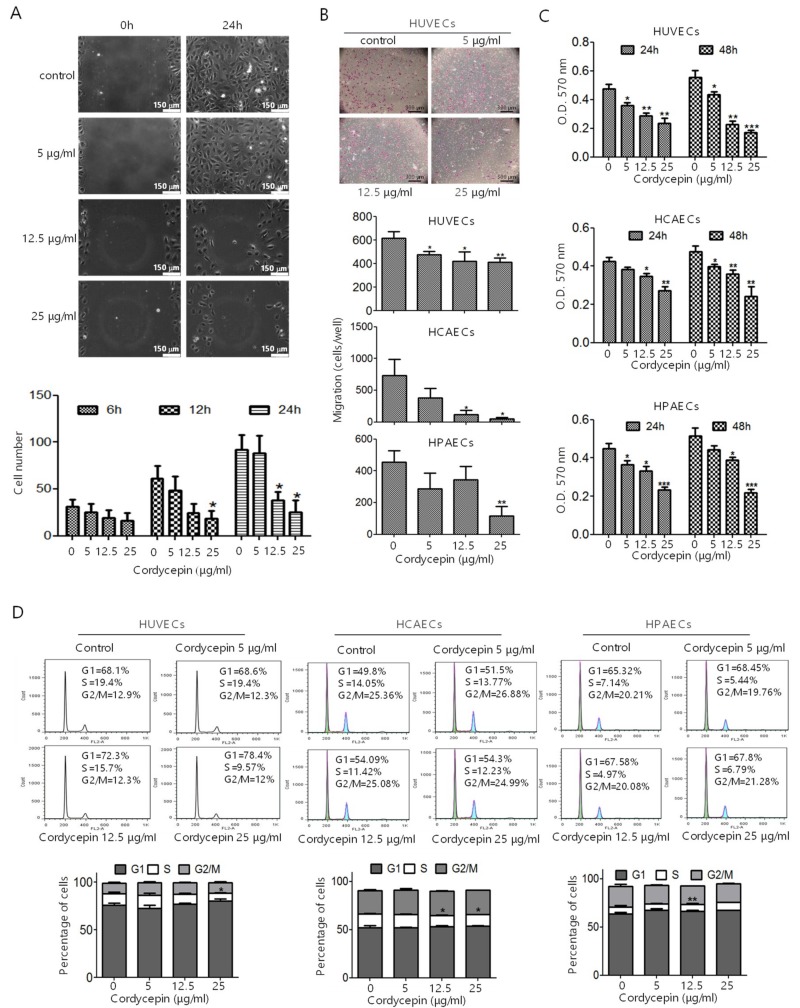
Inhibition of EC migration and proliferation by cordycepin. (**A**) HUVECs were treated with 0–25 μg/mL cordycepin for 24 h. EC migration was examined by wound healing assay. (**B**) HUVECs, HCAECs, and HPAECs were treated with 0–25 μg/mL cordycepin for 24 h. EC migration was examined by transwell chamber assay. (**C**) HUVECs, HCAECs, and HPAECs were treated with 0–25 μg/mL cordycepin for 24 h or 48 h. EC proliferation was determined by 3-(4,5-Dimethylthiazol-2-yl)-2,5-diphenyltetrazolium bromide) (MTT) assay. (**D**) HUVECs, HCAECs, and HPAECs were treated with 0–25 μg/mL cordycepin for 24 h. The cell cycle was examined by flow cytometry analysis. Scale bars: mean ± SD. *, *p <* 0.05; **, *p* < 0.01; ***, *p* < 0.001.

Wound healing assay was performed to characterize the effect of cordycepin on EC migration. HUVECs were seeded into silicone inserts and treated with different doses of cordycepin for 6, 12, and 24 h. Cordycepin suppressed the migratory activity of ECs in a dose-dependent manner (Figure 2A). Furthermore, HUVECs, HCAECs, and HPAECs were pre-treated with cordycepin and subjected to transwell chamber analysis. We found that treatment with cordycepin reduced the migration of ECs (Figure 2B). 

We determined whether cordycepin affects the proliferation and cell cycle of ECs. HUVECs, HCAECs, and HPAECs were treated with cordycepin for 24 h or 48 h and subjected to MTT assay. Cordycepin significantly inhibited the proliferation of HUVECs (Figure 2C, upper panel), HCAECs (Figure 2C, middle panel), and HPAECs (Figure 2C, lower panel) in a dose-dependent manner. We further examined the effect of cordycepin on cell cycle progression. HUVECs, HCAECs, and HPAECs were treated with cordycepin for 24 h and subjected to flow cytometry analysis. We found that cordycepin induced the cell cycle arrest of HUVECs by increasing the percentage of G1 phase cells (68.1% vs. 78.4% at 25 μg/mL) and reducing the percentage of S phase cells (19.4% vs. 9.57% at 25 μg/mL) (Figure 2D, left panel). Similar results can be found in HCAECs and HPAECs (Figure 2D, middle and right panels). 

We have previously reported that a proteasome inhibitors bortezomib (PS-341) suppresses FAK expression, thereby inducing apoptosis of cancer cells [31]. To elucidate whether cordycepin suppresses FAK expression correlates with induction of EC apoptosis, we examined the percentage of sub-G1 phase cells by flow cytometry and cleaved poly (ADP-ribose) polymerase (PARP) by western blotting analysis in HUVECs. PS-341 was used as positive control for the induction of apoptosis. We found that treatment of cordycepin (up to 25 μg/mL) has no significant on induction of apoptosis in ECs (Figure S2A,B).

**Figure 3:**
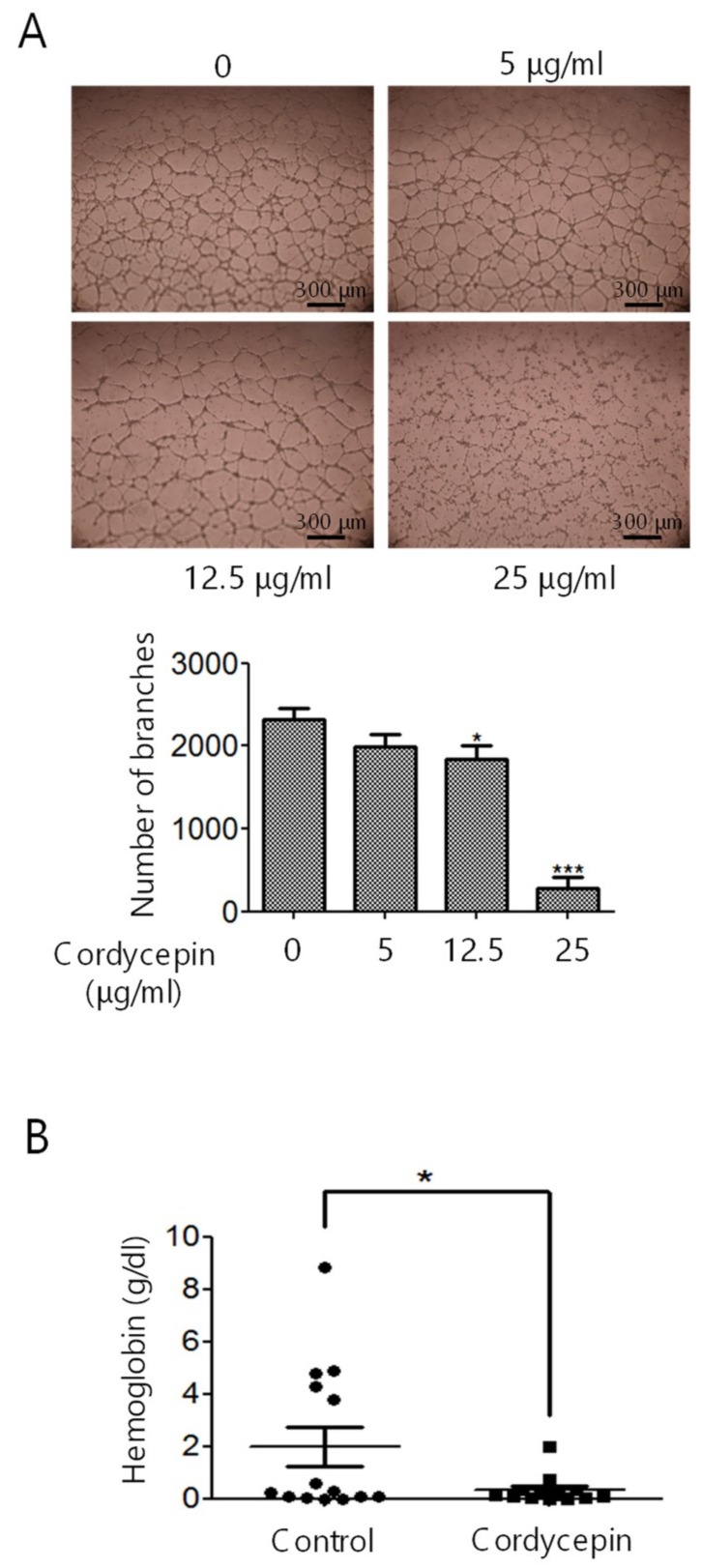
Suppression of tube formation and in vivo angiogenesis by cordycepin. (**A**) HUVECs were treated with cordycepin for 48 h and seeded onto Matrigel-coated plates with 0–25 μg/mL cordycepin in M200 medium for another 6 h. Tube formation was examined by counting the number of branches. (**B**) Matrigel plug assay was performed to assess in vivo angiogenesis formation in C57BL/6 mice. The mice were implanted with Matrigel plugs containing vascular endothelial growth factor (VEGF) and heparin with or without 25 μg/mL cordycepin for seven days. The Matrigel plugs were harvested, and in vivo angiogenesis was evaluated by measuring the concentration of hemoglobin. Scale bars: mean ± SD. *, *p* < 0.05; ***, *p* < 0.001.

For tube formation analysis, HUVECs were pre-treated with cordycepin for 48 h, and the cells were seeded onto Matrigel-coated plates for another 6 h with cordycepin. Cordycepin greatly impaired the network (Figure 3A, upper panel) and reduced tube formation, as demonstrated by the decreasing number of branches of HUVECs (Figure 3A, lower panel). 

In vivo Matrigel plug assay was performed to elucidate the effect of cordycepin on angiogenesis. Matrigel plugs containing VEGF and heparin were mixed with cordycepin and subcutaneously implanted into C57BL/6 mice for seven days. The Matrigel plugs were harvested, and in vivo angiogenesis was examined by measuring the level of hemoglobin. The results revealed that cordycepin significantly reduced the level of hemoglobin (Figure 3B).

### 2.3. Suppression of the Expression of FAK in ECs by Cordycepin

**Figure 4:**
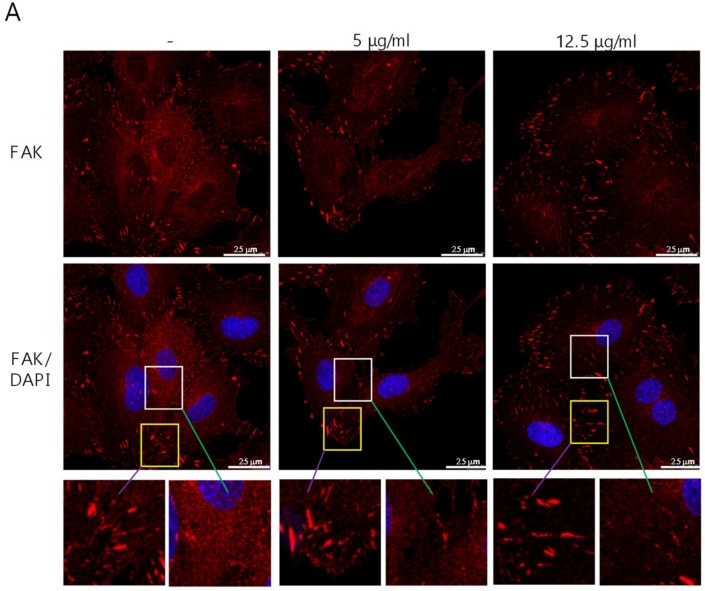

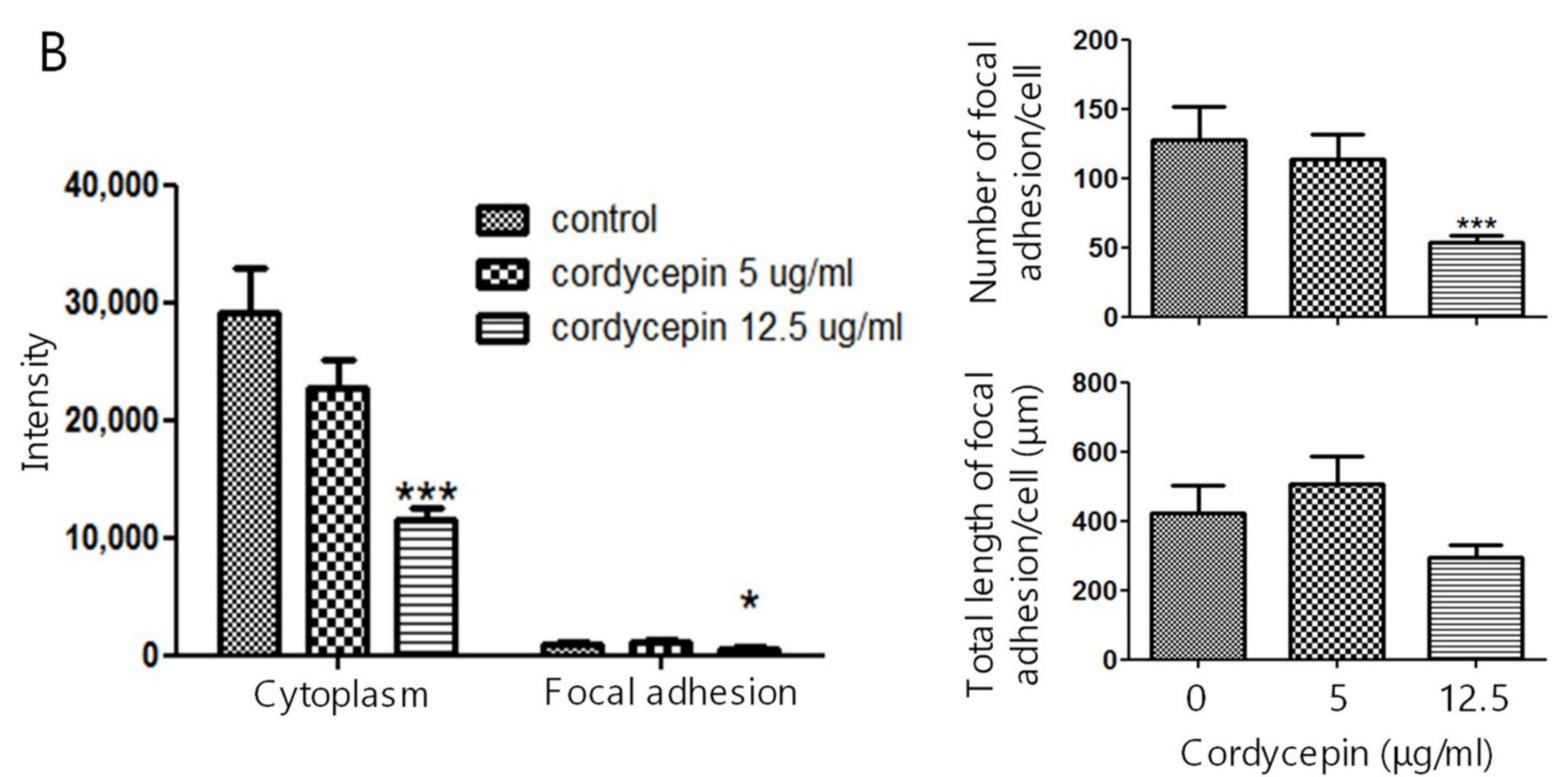
Suppression of the cytoplasmic expression of FAK in HUVECs by cordycepin. (**A**) HUVECs were treated with 0–12.5 μg/mL cordycepin for 24 h. The expression level of FAK was determined by confocal microscopy. (**B**) FAK expression was quantified based on staining intensity, and number and total length of focal adhesion were determined by using MetaMorph software (Version 7.10.1.161, Leica, Wetzlar, Germany). Scale bars: mean ± SD. *, *p* < 0.05; ***, *p* < 0.001.

To elucidate the suppressive effect of cordycepin on FAK in ECs, HUVECs were treated with cordycepin for 24 h. The subcellular localization and expression level of FAK were examined by fluorescence confocal microscopy. We found that FAK was mainly expressed in the cytoplasm and focal adhesions, and a lower level of FAK was detected in the nucleus (Figure 4A, left and upper panel). Cordycepin significantly reduced the level of FAK expression (Figure 4A, upper, middle, and right panels). Interestingly, we found that treatment with cordycepin at 12.5 μg/mL significantly inhibited FAK expression in the cytoplasm and focal adhesions (Figure 4B, left panel), reduced focal adhesion number but only slightly decreased length of focal adhesion of HUVECs (Figure 4B, right panels). 

### 2.4. Induction of p53 and p21 Expression in ECs by Cordycepin

**Figure 5:**
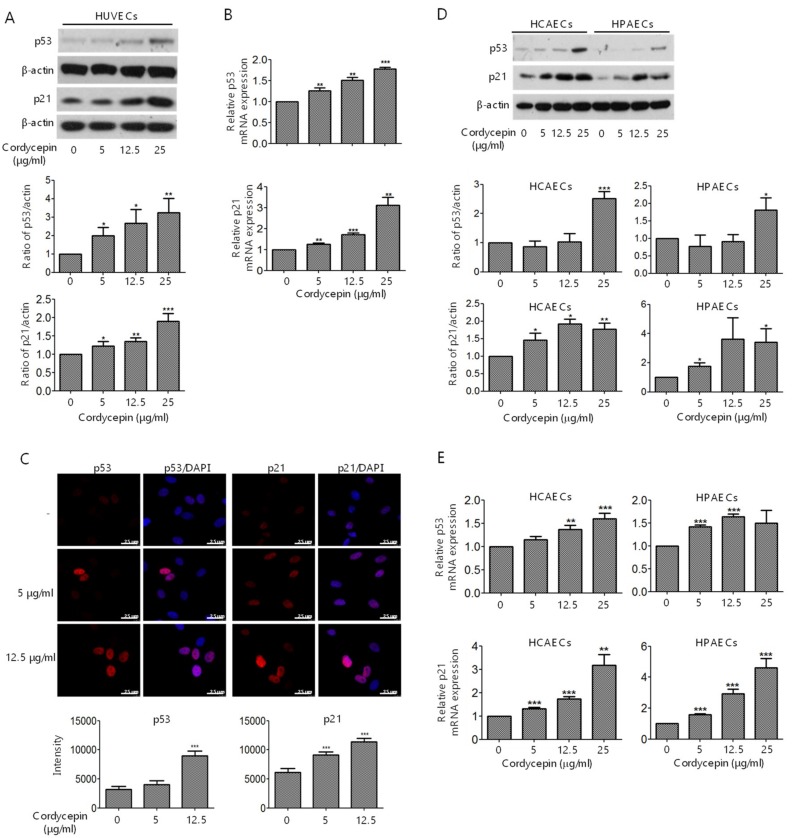
Induction of p53 and p21 expression in ECs by cordycepin. (**A**,**D**) HUVECs, HCAECs, and HPAECs were treated with 0–25 μg/mL cordycepin for 24 h. The expression levels of p53 and p21 were determined by western blotting analysis. (**B**,**E**) HUVECs, HCAECs, and HPAECs were treated with 0–25 μg/mL cordycepin for 4 h. The expression levels of p53 and p21 were determined by qPCR. (**C**) HUVECs were treated with 0–12.5 μg/mL cordycepin for 24 h. The expression levels of p53 and p21 were determined by confocal microscopy. β-actin was used as the loading control. Scale bars: mean ± SD. *, *p* < 0.05; **, *p* < 0.01; ***, *p* < 0.001.

Previous studies have demonstrated that FAK directly interacts with p53 and regulates p53 stability [14,15,32]. We hypothesized that cordycepin may suppress FAK expression and consequently promote p53 stability and nuclear translocation and activation. We first confirmed the expression of p53 and the downstream factor p21 in HUVECs treated with various concentrations of cordycepin. Cordycepin induced p53 and p21 expression in a dose-dependent manner in HUVECs, as demonstrated by western blotting (Figure 5A) and qPCR (Figure 5B) analysis. Moreover, the expression and nuclear translocation of p53 and p21 were examined by fluorescence confocal microscopy. Cordycepin significantly promoted the accumulation and nuclear translocation of p53 and p21 in HUVECs (Figure 5C). To further confirm the effect of cordycepin on the induction of p53 and p21 in ECs, HCAECs and HPAECs were treated with cordycepin, and the expression levels of p53 and p21 were determined by western blotting and qPCR. The results revealed that cordycepin induced p53 and p21 expression in both HCAECs and HPAECs (Figure 5D,E).

### 2.5. Inhibition of the Proliferation and Tumor Growth of HCC Cells by Cordycepin

**Figure 6:**
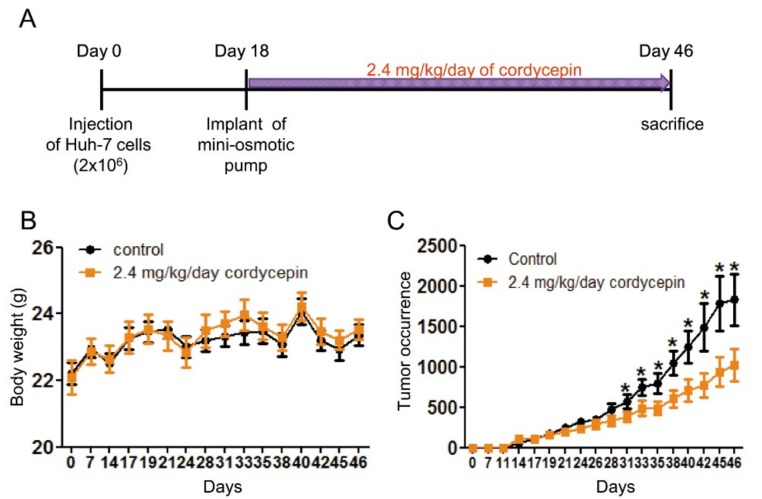
Reduction of in vivo HCC tumor growth by cordycepin. (**A**) An outline of the experimental procedure. Micro-osmotic pumps delivering either the control (DMSO) or cordycepin were implanted subcutaneously into nude mice 18 days after the injection of Huh-7 cells. The (**B**) body weight and (**C**) tumor volume of nude mice (*n* = 9) were assessed. *, *p* < 0.05.

Tumor growth in HCC is highly dependent on angiogenesis [33]. As cordycepin could suppress the in vitro tube formation of ECs and in vivo angiogenesis, we next investigated whether cordycepin attenuates the in vivo tumor growth of HCC cells. We first evaluated the expression of FAK and the suppressive effect on cell proliferation. We found that cordycepin suppressed FAK and p-FAK expression in Huh-7 cells (Figure S3). In addition, we found that cordycepin inhibited the proliferation of Huh-7, HepG2, and Hep3B cells, as demonstrated by MTT assay (Figure S4). To investigate in vivo HCC tumor growth, Huh-7 cells were subcutaneously injected into nude mice. Micro-osmotic pumps containing DMSO or cordycepin (2.4 mg/kg/day) were implanted subcutaneously into nude mice on day 18 (Figure 6A). The results revealed that cordycepin did not have a significant effect on body weight (Figure 6B). However, cordycepin significantly reduced tumor size (Figure 6C).

## 3. Discussion

FAK plays a crucial role in regulating the cellular functions of ECs and the angiogenesis process [6,7,8,9]. FAK is overexpressed in various types of malignancies [34] and a recent study indicated that knockout or functional mutation of FAK result in attenuating cell invasion into dense 3D matrices [35]. Moreover, we have previously reported that the overexpression of FAK is associated with a higher incidence of extrahepatic metastasis and poorer survival in HCC [36]. Our previous study revealed that cordycepin could suppress the EMT and the expression of FAK, integrin α3, integrin α6, and integrin β1 in HCC [17]. However, whether cordycepin modulates FAK expression and angiogenesis in ECs has not been determined. Here, we showed for the first time that cordycepin suppressed FAK expression and phosphorylation in HUVECs, HCAECs, and HPAECs (Figure 1A–D). Expression of p53 can be induced in response to DNA damage and p21 (cyclin-dependent kinase inhibitor) is upregulated by p53 and contribute into modulating G1 cell cycle arrest [37,38,39]. Results from several studies indicated that cordycepin induces p53 in various cancer cells [28,40,41,42]. However, whether cordycepin regulates p53/p21 axis in ECs has never been investigated. Here, we demonstrated that cordycepin reduced FAK expression (Figure 4) and increased nuclear p53/p21 accumulation in ECs (Figure 5). As a result, cordycepin reduced EC migration (Figure 2A,B), EC proliferation (Figure 2C), EC tube formation (Figure 3A), in vivo angiogenesis (Figure 3B), and HCC tumor growth (Figure 6B). 

The expression of FAK is regulated by the activation of NFκB and downregulated by p53 [10]. Several studies have reported that cordycepin induces p53 [28,41], and FAK deletion promotes p53-mediated p21 induction, the DNA damage response, and radio-resistance [43]. However, the molecular mechanism of how cordycepin induces p53 remains unclear. Our current results demonstrated the suppression of FAK expression in ECs by cordycepin, which may explain how cordycepin induces p53 and p21 expression as well as suppresses angiogenesis and tumor growth. 

Cordycepin is an analog of adenosine and has a short in vivo half-life in mice because it can be degraded by adenosine deaminase [9,44]. To maintain the effect of cordycepin in the xenograft mice experiment, we performed an in vivo study by implanting micro-osmotic pumps in HCC tumor-bearing mice (Figure 6B). We found that the stable release of cordycepin had a significant effect on the suppression of tumor growth. This may help devise a potential strategy to address stability concerns when using cordycepin as an anti-tumor compound in future applications.

HCC is a highly vascular malignancy, and angiogenesis plays a crucial role in HCC tumor progression [33]. An earlier study indicated that cordycepin could exert a potential anti-angiogenic effect of EA.hy926 cells [45]. A relatively high concentration (125 μg/mL) of cordycepin was used, possibly because EA.hy926 cells are a hybrid of HUVECs and lung adenocarcinoma A549 cells [45]. In our study, we demonstrated that cordycepin suppressed the proliferation (Figure 2C,D), migration (Figure 2A,B), and tube formation (Figure 3A,B) of primary cultured ECs (HUVECs, HCAECs, and HPAECs) at physiologically reasonable concentrations (5–25 μg/mL). We also showed that cordycepin reduced FAK expression (Figure 1A–D) and induced p53/p21 expression (Figure 5A–E) in ECs. These results support the anti-angiogenic effect of cordycepin.

The process of angiogenesis contributes to tumor growth and progression. To obtain an adequate supply of oxygen and nutrients, cancer cells can induce the proliferation and migration of ECs to form new blood vessels. FAK is overexpressed in various types of cancers [2,34] and plays a crucial role in regulating the angiogenesis process [5,6,7,8,9]; thus, targeting FAK may be a potential therapeutic approach in cancer therapy. A cell-penetrating peptide of 28 amino acids, derived from the bacterial toxin azurin, has been reported to inhibit angiogenesis and tumor growth by impairing the phosphorylation of FAK, Akt, and VEGFR-2, consequently inhibiting the motility and migration of HUVECs [46]. Moreover, a small molecular compound that directly disrupts the FAK-p53 interaction has been developed to reactivate p53 and block tumor growth [47]. In this study, we showed that regulation of the FAK/p53/p21 axis by cordycepin resulted in the attenuation of EC proliferation, migration, and angiogenesis. We also demonstrated that cordycepin suppressed FAK in cancer cells, thereby reducing cell proliferation and HCC tumor growth. We further examined the number of ECs in the tumor regions of tumor-bearing mice. However, quantification of the number of ECs or vessels was not possible due to severe necrosis in the tumor regions (Figure S5). 

## 4. Materials and Methods 

### 4.1. Cell Culture and Reagents

HUVECs were purchased from the American Type Culture Collection (ATCC; Manassas, VA, USA), and HCAECs and HPAECs were obtained from Lonza (Walkersville, MD, USA). HUVECs, HCAECs, and HPAECs were maintained in culture media as described in Table S1. The HCC cell lines Huh-7 (Japanese Collection of Research Bioresources, Osaka, Japan), HepG2, and Hep3B (ATCC) were cultured in Dulbecco’s modified Eagle’s medium supplemented with 10% FBS (Hyclone; Thermo Fisher Scientific, Waltham, MA, USA). Cordycepin (purity 98.0%) was purchased from Sigma-Aldrich (St. Louis, MO, USA).

### 4.2. Western Blot Analysis

Cells treated with the indicated concentrations of cordycepin were incubated with RIPA lysis buffer (Millipore, Billerica, MA, USA) containing protease and phosphatase inhibitor cocktails. Supernatant of cell lysate was collected and 30 μg of total protein from each sample was loaded onto a gradient SDS-PAGE gel and subsequently transferred to PVDF membranes. The membranes were blocked and hybridized with primary antibodies against FAK, phosphorylated FAK (p-FAK) at Tyr397, p53, p21, and β-actin (Table S2). Then, the membranes were incubated with secondary antibodies, and immunoreactive bands were visualized by X-ray film exposure. Protein expression levels were quantified by densitometric analysis. 

### 4.3. qPCR Analysis

The total RNA of cordycepin-treated ECs was extracted using RNA_ZOL_^®^ RT (Molecular Research Center Inc., Cincinnati, OH, USA), and cDNA was synthesized using PrimeScript^TM^ RT Reagent Kit (TaKaRa, Otsu, Japan). Relative RNA expression levels were detected by qPCR (LightCycler 480II; Roche, Penzberg, Germany) using KAPA SYBR^®^ FAST qPCR Kit (Kapa Biosystems, Wilmington, MA, USA) with specific primers for human FAK, p53, and p21 (Table S3). The levels of gene expression were normalized to GAPDH expression.

### 4.4. Cell Migration and Wound Healing Analysis

HUVECs, HCAECs, and HPAECs were pretreated with cordycepin in 1% FBS medium for 24 h and loaded into polycarbonate transwells (BD, San Diego, CA, USA). The upper chamber contained 1 × 10^4^ cells incubated with cordycepin in medium containing FBS (HUVECs, 1%; HCAECs, 0.5%; HPAECs, 0.2%), and the lower compartment was filled with culture medium containing cordycepin and FBS (HUVECs, 10%; HCAECs, 5%; HPAECs, 2%). After 4 h, migratory cells were fixed with 4% paraformaldehyde and stained with crystal violet. Cell migration was quantified by counting the number of migrated cells using a microscope. For wound healing assay, 1 × 10^5^ HUVECs were seeded into silicone inserts (Culture-Inserts; ibidi GmbH, Martinsried, Germany) and incubated with M200 medium containing 1% FBS and the indicated concentrations of cordycepin. The number of migrated cells was calculated using a microscope (AF6000; Leica, Wetzlar, Germany).

### 4.5. Cell Proliferation Analysis

Cell proliferation was analyzed by MTT assay. Cells were seeded into 96-well plates and incubated with 1% FBS medium (Hyclone; Thermo Fisher Scientific, Waltham, MA, USA) containing the indicated concentrations of cordycepin for 24 h or 48 h. MTT was added to each well and incubated at 37 °C for 3 h. Subsequently, the yellow MTT solution was removed, and 200 μL of dimethyl sulfoxide was added. The absorbance at 570 nm was measured with a reference wavelength of 690 nm.

### 4.6. Flow Cytometry Analysis

HUVECs, HCAECs, and HPAECs treated with cordycepin for 24 h were harvested, fixed with ethanol, washed with ice-cold PBS, and subsequently incubated with propidium iodide. The cell cycle distribution was analyzed using FACS Calibur (BD, San Jose, CA, USA).

### 4.7. Tube Formation

HUVECs (1.4 × 10^5^) were pretreated with cordycepin in 1% FBS medium for 48 h, re-seeded into 24-well plates pre-coated with 200 μL/well Matrigel (Corning, Bedford, MA, USA), and incubated at 37 °C for 30 min. Then, cells in the Matrigel-coated 24-well plates were incubated with M200 medium supplemented with 1% FBS and cordycepin. After incubation for 6 h, the tube formation of ECs was examined by counting the number of branches at every five random fields using a microscope.

### 4.8. Immunofluorescence Confocal Microscopy

HUVECs were seeded onto glass coverslips for 24 h, followed by treatment with cordycepin for another 24 h. Cells were fixed with 2% paraformaldehyde, permeabilized with 0.1% Triton X-100 in PBS, and blocked with 10% FBS, followed by incubation with specific antibodies for FAK, p53, and p21. Then, the cells were washed with 0.02% Triton X-100, labeled with secondary antibodies, and mounted in DAPI Fluoromount-G solution (Southern Biotech, Birmingham, AL, USA). The expression levels and subcellular localization of FAK, p53, and p21 were examined by confocal microscopy (Leica). Number and length of focal adhesions, FAK, p53, and p21 expression levels in ten random fields (34–53 cells) were quantified using MetaMorph software (Version 7.10.1.161, Leica, Wetzlar, Germany).

### 4.9. In Vivo Matrigel Plug Angiogenesis Assay and Tumor Xenograft Experiments

The protocols of this study were approved by the Institutional Animal Care and Use Committee of the National Health Research Institutes, Taiwan. C57BL/6 and BALB/c nude mice were purchased from the National Laboratory Animal Center, Taiwan. C57BL/6 mice were implanted with 250 μL Matrigel (Corning) containing 100 ng/mL VEGF (Sigma-Aldrich) and 20 U/mL heparin (B. Braun Melsungen AG, Melsungen, Germany) with or without 25 μg/mL cordycepin for seven days. The level of hemoglobin was measured using Drabkin’s reagent (Sigma-Aldrich). For in vivo tumor xenograft experiments, BALB/c nude mice were subcutaneously injected with 2 × 10^6^ Huh-7 cells. Micro-osmotic pumps (ALZA; Palo Alto, CA, USA) with in vivo continuous delivery [48,49] containing DMSO or cordycepin (2.4 mg/kg/day) were implanted subcutaneously into nude mice on day 18. Tumor volume was determined by sequential caliper measurement of the length (L) and width (W), calculated as LW^2^/2.

## 5. Conclusions

Taken together, our findings demonstrated the dual role of cordycepin in regulating FAK and p53 signaling in ECs and cancer cells via anti-angiogenic and anti-tumor activity. As antiangiogenic monotherapy may not be sufficient to eradicate tumor progression, cordycepin may be used as a potential adjuvant or supplement for cancer therapy to suppress tumor angiogenesis and tumor growth. Further investigation is needed to elucidate the effect of cordycepin synergized with other anti-tumor therapy.

## Supplementary Materials

The following are available online at http://www.mdpi.com/2072-6694/11/2/168/s1, Figure S1: Structure of cordycepin and adenosine, Figure S2: Cordycepin has no significant effect on inducing HUVEC apoptosis, Figure S3: Cordycepin suppresses FAK expression and phosphorylation in HCC, Figure S4: Cordycepin inhibits cell proliferation of HCC, Figure S5: Representative immunohistochemistry staining of CD31 in tumors of xenograft nude mice treated without or with cordycepin, Table S1: Culture medium of HUVECs, HCAECs and HPAECs, Table S2: Antibodies used in this study; Table S3: Primer sequences for Q-PCR used in this study.

## Abbreviations

ECs	endothelial cells
EMT	epithelial–mesenchymal transition
FAK	focal adhesion kinase
p-FAK	FAK phosphorylation at Tyr397
HCAECs	human coronary artery endothelial cells
HCC	hepatocellular carcinoma
HPAECs	human pulmonary artery endothelial cells
HUVECs	human umbilical vein endothelial cells
MTT	3-(4,5-dimethylthiazol-2-yl)-2,5-diphenyltetrazolium bromide
Q-PCR	quantitative real-time PCR.
