# Supplementary material for: Cordycepin Suppresses Endothelial Cell Proliferation, Migration, Angiogenesis, and Tumor Growth by Regulating Focal Adhesion Kinase and p53
Source: Cancers (Basel). 2019 Feb 1;11(2):168. doi: 10.3390/cancers11020168 (PMC6406613; doi:10.3390/cancers11020168)
Supplement: Supplementary file 1 [file cancers-11-00168-s001.pdf]

Supplementary Materials: Cordycepin Suppresses Endothelial Cell Proliferation, Migration, Angiogenesis, and Tumor Growth by Regulating Focal Adhesion Kinase and p53

Yi-Ting Lin, Shu-Man Liang, Ya-Ju Wu, Yi-Ju Wu, Yi-Jhu Lu, Yee-Jee Jan, Bor-Sheng Ko, Yung-Jen Chuang, Song-Kun Shyue, Cheng-Chin Kuo and Jun-Yang Liou

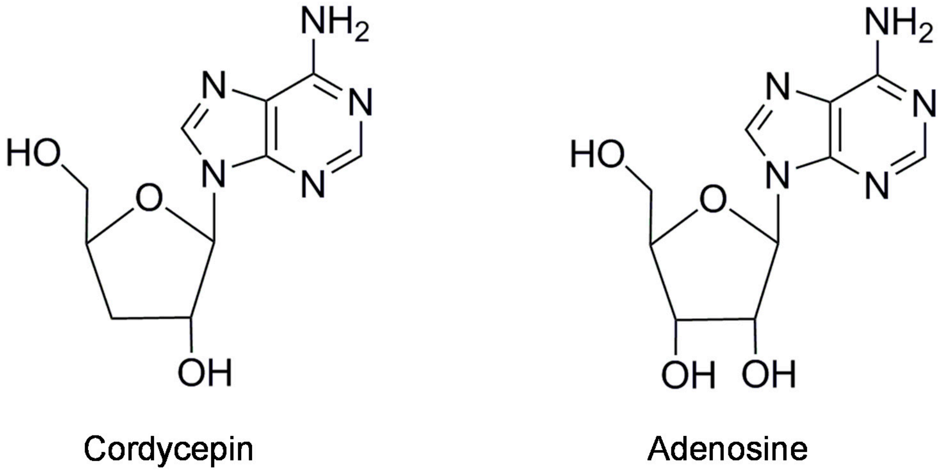

Figure S1. Structure of cordycepin and adenosine.

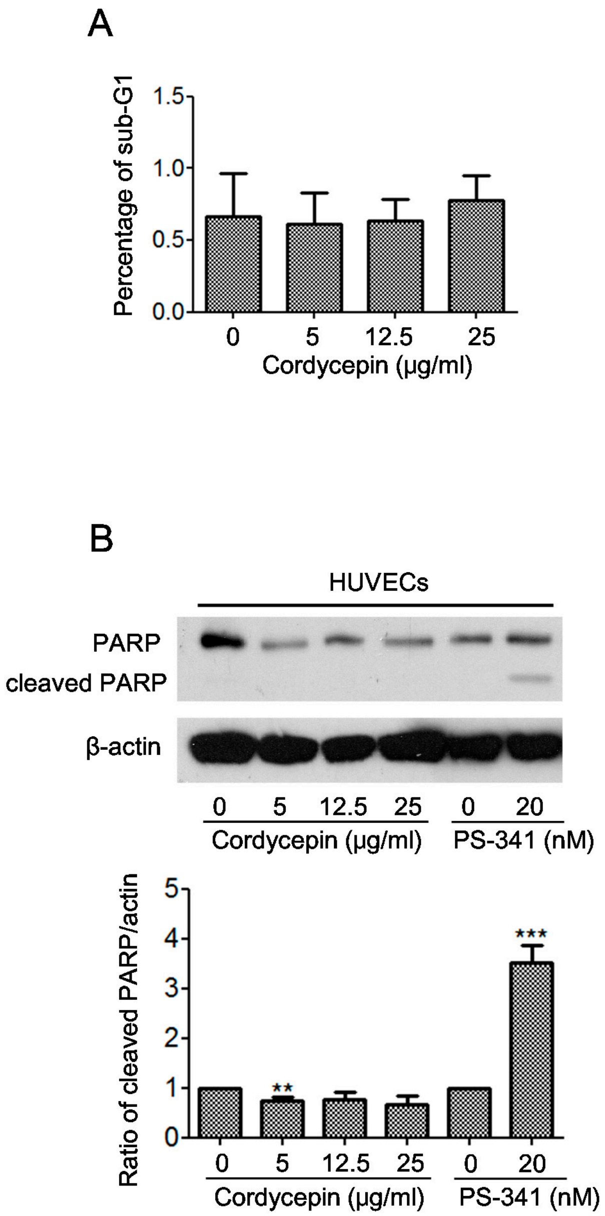

Figure S2. Cordycepin has no significant effect on inducing HUVEC apoptosis. (**A**) HUVECs were treated with 0–25 μg/mL cordycepin for 24 h. The percentage of sub-G1 cells was examined by flow cytometry analysis. Scale bars: mean ± SD. (**B**) HUVECs were treated with 0–25 μg/mL cordycepin and 5 ng/mL PS-341 (bortezomib) for 24 h. Expression of cleaved PARP was determined by Western blotting analysis. β-actin was used as loading control. ** $p < 0.01$, *** $p < 0.005$

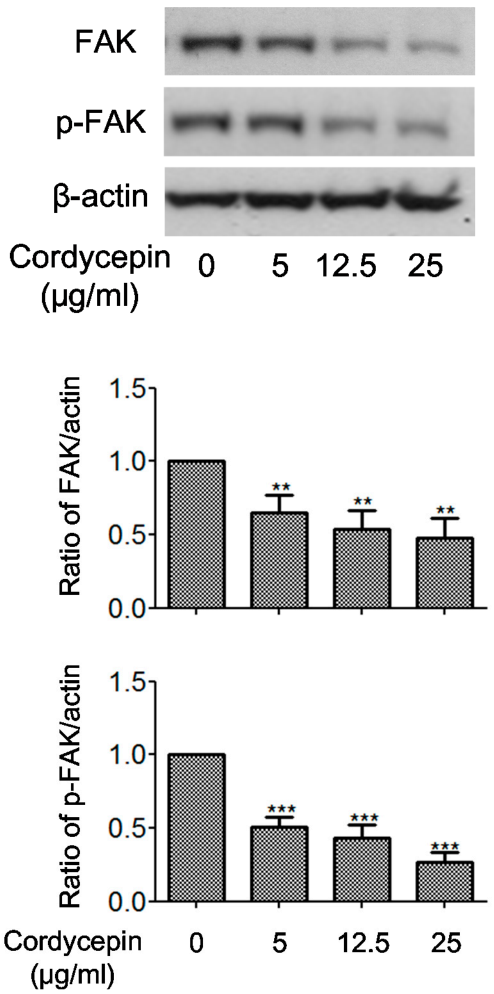

Figure S3. Cordycepin suppresses FAK expression and phosphorylation in HCC. Huh-7 cells were treated with 0–25 μg/mL cordycepin for 24 h. Expressions of FAK and p-FAK were determined by Western blotting analysis. β-actin was used as loading control. ** $p < 0.01$, *** $p < 0.005$

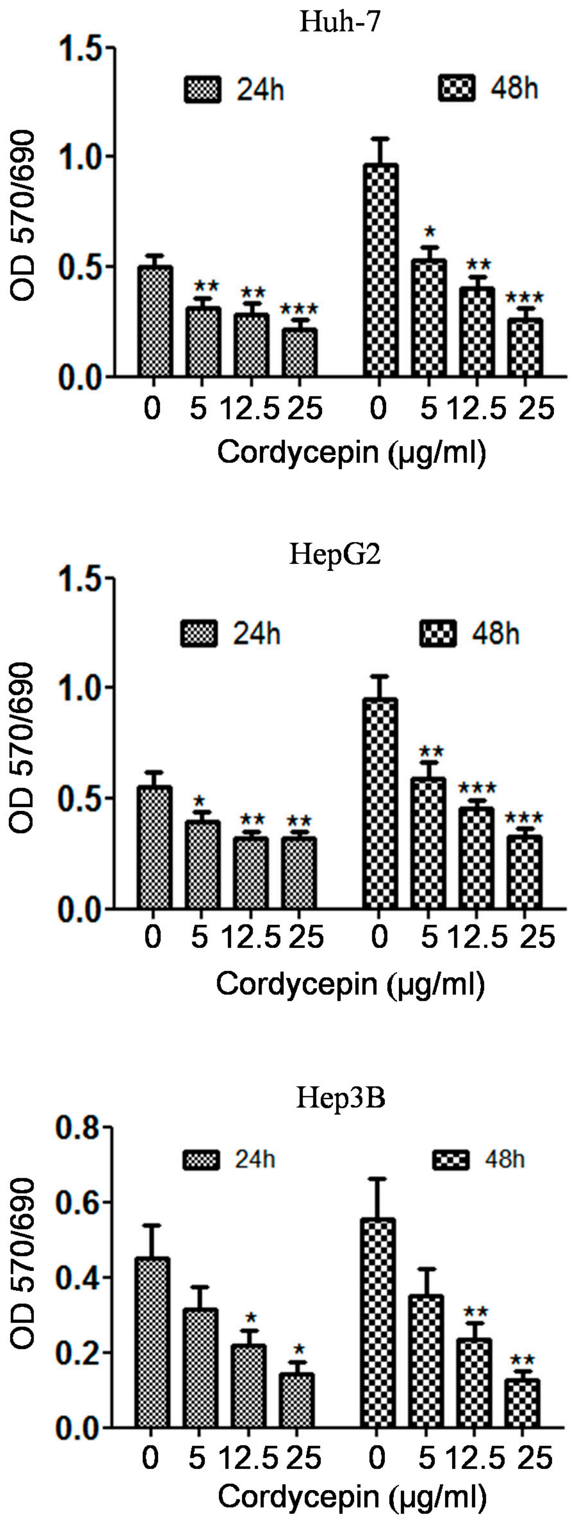

Figure S4. Cordycepin inhibits cell proliferation of HCC. Huh-7, HepG2 and Hep3B cells were treated with 0–25 μg/mL cordycepin for 24 h or 48 h. Cell viability was determined by MTT assay. Scale bars: mean ± SD. *, $p < 0.05$; **, $p < 0.01$; ***, $p < 0.001$.

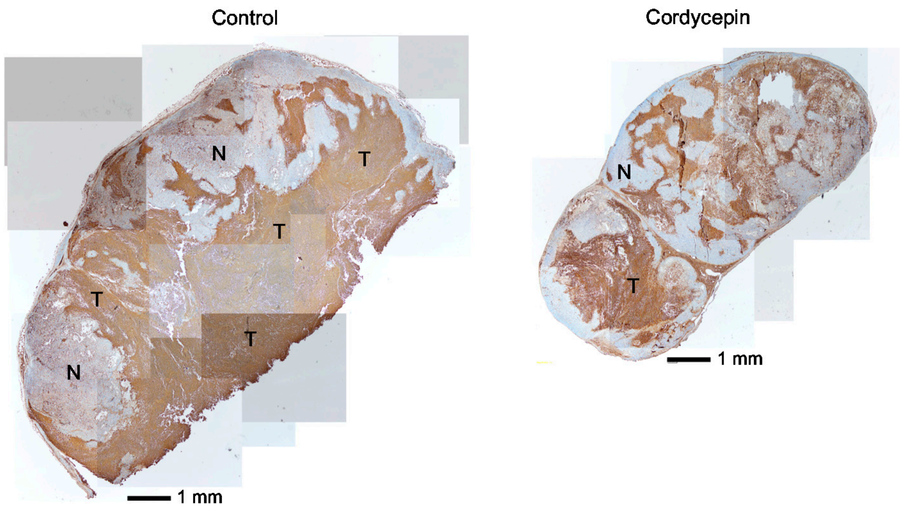

Figure S5. Representative immunohistochemistry staining of CD31 in tumors of xenograft nude mice treated without (control) or with cordycepin (2.4 mg/kg/day). T: tumor; N: necrosis region.

Table S1. Culture medium of HUVECs, HCAECs and HPAECs.

Cells	Culture Medium
HUVECs	M200 medium (Gibco, Gaithersburg, MD, USA) supplemented with 10% fetal bovine serum (FBS) (Biological Industries, Kibbutz BeitHaemek, Israel), 100 unit/mL penicillin, 100 mg/mL streptomycin and low serum growth supplement (LSGS) (Gibco).
HCAECs	Endothelial cell growth medium (EGM™-2 MV) medium supplemented with 5% FBS, 0.04% hydrocortisone, 0.4% hFGF-B, 0.1% VEGF, 0.1% R3-IFG-1, 0.1% ascorbic acid, 0.1% hEGF, 0.1% gentamicin and amphotericin-B (GA-1000) (Lonza, Walkersville, MD, USA).
HPAECs	Endothelial cell growth medium-2 (EGM™-2) medium supplemented with 2% FBS, 0.04% hydrocortisone, 0.4% hFGF-B, 0.1% VEGF, 0.1% R3-IFG-1, 0.1% ascorbic acid, 0.1% hEGF, 0.1% GA-1000, 0.1% heparin (Lonza).

Table S2. Antibodies used in this study.

Antibodies	Dilution
FAK	1:5000 (Santa Cruz, Dallas, TX, USA)
p-FAK (Tyr397)	1:1000 (GeneTex, Hsinchu, Taiwan)
p53	1:500 (Cell signaling, Danvers, MA, USA)
p21	1:500 (Cell signaling)
-actin	1:5000 (Sigma-Aldrich, Saint Louis, MO, USA)
PARP	1:1000 (Cell signaling, Danvers, MA, USA)

Table S3. Primer sequences for Q-PCR used in this study.

Gene	Oligonucleotide Primer
FAK	Forward: TCCCTATGGTGAAGGAAGTC<br>Reverse: TTCTGTGCCATCTCAATCTC
p53	Forward: ATTTGCGTGTGGAGTATTTGGATGA<br>Reverse: GTAGTGGATGGTGGTACAGTCAGA
p21	Forward: AGACTCTCAGGGTCGAAAAC<br>Reverse: TAAGGCAGAAGATGTAGAGC
GAPDH	Forward: ACCACAGTCCATGCCATCACTG<br>Reverse: GTTCAGCTCAGGGATGACCTTG

© 2019 by the authors. Licensee MDPI, Basel, Switzerland. This article is an open access article distributed under the terms and conditions of the Creative Commons Attribution (CC BY) license (http://creativecommons.org/licenses/by/4.0/).
